# ECG changes in patients with primary hyperthyroidism

**DOI:** 10.11604/pamj.2018.30.246.12244

**Published:** 2018-08-02

**Authors:** Ishtiaque Hussain Baladi, Ayesha Aslam Rai, Syed Masroor Ahmed

**Affiliations:** 1 Department of Medicine, Medical Unit III, Ward 7, Jinnah Postgraduate Medical Centre, Karachi, Pakistan

**Keywords:** Cardiovascular, electrocardiographic, hyperthyroidism

## Abstract

**Introduction:**

Thyroid hormones plays key role in regulating cardiovascular system. Its imbalance leads to various electrophysiological changes in cardiovascular system. This study was done to determine the frequency of electrocardiographic (ECG) changes in patients with primary hyperthyroidism.

**Methods:**

It was a descriptive cross-sectional study conducted in the Department of Medicine, Medical Unit III, Ward-7, Jinnah Postgraduate Medical Centre, Karachi, from October 2013 to April 2014. A total of 103 patients newly diagnosed with primary hyperthyroidism were included in this study. Venous blood samples were collected for T_3_, T_3_, TSH analyzed by radioimmunoassay. ECG was performed. Outcome variables were the ECG changes i.e. sinus tachycardia and atrial fibrillation.

**Results:**

The average age of the patients was 30.09±5.57 years (95%CI: 29 to 31.18). Out of 103 cases, 19 (18.45%) were male and 84 (81.55%) were female. Sinus tachycardia was observed in 60.19% (62/103) patients whereas atrial fibrillation was found in 11.65 (12/103) of cases.

**Conclusion:**

In this study frequency of electrocardiographic changes in term of sinus tachycardia was high. This report has emphasized the importance of thyrotoxicosis as a cause of cardiac morbidity and mortality in patients with thyrotoxicosis. These cardiac complications are readily reversible if timely optimal treatment is offered.

## Introduction

The cardiovascular system is very sensitive to thyroid hormone [1]. Thyroid hormone exerts both electrophysiological and ionotropic effects on the heart and may increase the risk of developing atherosclerosis [2]. Patients with hyperthyroidism can develop atrial arrhythmias such as sinus tachycardia, atrial flutter, atrial fibrillation, both prolonged and shortened QT interval [2]. It is also linked with angina, rarely myocardial infarction and sudden death [3]. Hyperthyroidism results in systemic cardiovascular hemodynamic changes [1] which is associated with enhanced sympatho-adrenal activity due to increase adrenergic receptor number and sensitivity, consequently leads to coronary artery spasm [1]. Clinical signs and symptoms suggestive of diagnosis include type of chest pain, palpitation, weight loss, diaphoresis and agitation [4]. Incidence of cardiac disease in thyrotoxicosis is 27% with male to female ratio of 1:5 [5], frequency of hypertension 53%, arrhythmia 25% and heart failure 42% [6]. Angina has been reported in 20% of the patients with thyrotoxicosis [4]. Several studies have reported that prolong QT interval is associated with increased risk of ventricular dysarrhythmia and sudden death [4]. Heart is one of the main target organs of thyroid hormone and its morphology and function are investigated by means of standard 12-leads ECG [7].

Generally, the prognosis for hyperthyroidism-associated coronary vasospasm is good [1]. Antithyroid drugs remained cornerstone in the management of hyperthyroidism [1]. Recognition and proper interpretation of ECG changes in hyperthyroidism is helpful in diagnosis, prognosis and therapy of hyperthyroidism [8]. Napoli et al. reported that a reasonable treatment approach might be to continue nitrates and/or calcium channel blocker like diltiazem, for 6-12 months after a euthyroid state is achieved [1, 9]. The purpose of this study is to identify electrocardiographic (ECG) changes in patients with primary hyperthyroidism in order to diagnose, treat and avoid morbidity and mortality related to cardiovascular system. To date no study has been done in our country on patients having cardiac disease secondary to hyperthyroidism.

## Methods

This descriptive cross-sectional study with non-probability purposive sampling was conducted at Medical Unit III ward 7, Jinnah Postgraduate Medical Centre (JPMC) Karachi from October 2013 to April 2014 after reviewed by Ethical Review Committee of JPMC Karachi. A total of 103 patients between ages 20-40 years regardless of gender visiting out-patient department of ward 7 were enrolled who were recently diagnosed with primary hyperthyroidism. Subjects with presence of all the following levels of thyroid hormones (T_3_ > 6.5 pmol/L, T_4_>20 pmol/L, TSH < 0.5 mIU/L) was considered as primary hyperthyroidism. The duration of diagnosis was taken less than a week. Patients already taking antithyroid drugs, e.g: propylthyuracil, carbimazole, methimazole, subjects with actual and impending thyrotoxic crisis, pregnant and lactating mothers were excluded from study. Patients with previously known co morbids like ischemic heart disease or with certain chronic illness like diabetes mellitus (FBS>100mg/dl), renal failure (serum creatinine >1.4 mg/dl) were also excluded from the study to eliminate bias.

After taken the informed consent, detailed history, examination and investigation was done to rule out exclusion criteria. Venous blood samples were collected for T_3_, T_3_, TSH analysed by radioimmunoassay. ECG was performed using Nihon Kohden device, model # 9 > 620L, 2005. Proforma was used for data collection and single laboratory was use to prevent laboratory bias. Outcome variables were ECG changes including sinus tachycardia (heart rate > 100 beat/min) and Atrial fibrillation (absent P-wave, irregular heart rate) [6]. Data was entered and analyzed in statistical software (SPSS-17). Frequencies and percentages were computed for qualitative variables like gender and ECG changes in primary hyperthyroidism. Mean ± SD were computed for quantitative variables like age. Effects modified were controlled by stratification of age, gender to see the impact of outcome. Post stratification chi-square test was applied. P < 0.05 was considered as significant.

## Results

A total of 103 patients newly diagnosed with primary hyperthyroidism were included in this study. The average age of the patients was 30.09±5.57 years (95%CI: 29 to 31.18) as shown in Table 1. Out of 103 cases, 19(18.45%) were male and 84(81.55%) were female. Frequency of electrocardiographic in term of sinus tachycardia and atrial fibrillation in patients with primary hyperthyroidism are presented in Table 2. Sinus tachycardia was observed in 60.19% (62/103) patients and atrial fibrillation was 11.65(12/103) cases. Rate of sinus tachycardia was observed above 50% cases in all age groups which is not statistically significant among different age groups (p = 0.68) as shown in Table 2 while rate of atrial fibrillation was significantly high in above 30 years of age patients (p=0.02) as presented in Table 2. Similarly rate of sinus tachycardia and atrial fibrillation was not statistically significant between male and female as presented in Table 1.

**Table 1:** Frequency of electrocardiographic changes in patients with primary hyperthyroidism with respect to age groups

Age (years)	Sinus tachycardia		Atrial fibrillation		Total (n=103)
	Yes	No	Yes	No	
<25	13 (54.2%)	11 (45.8%)	1 (4.2%)	23 (95.8%)	24 (23.3%)
26-30	19 (63.3%)	11 (36.7%)	1 (3.3%)	29 (96.7%)	30 (29.13%)
31-35	13 (54.2%)	11 (45.8%)	3 (12.5%)	21 (87.5%)	24 (23.30%)
36-40	17 (68%)	8 (32%)	7 (28%)	18 (72%)	25 (24.27%)
X^2^	1.48		9.83		
**p-value**	0.68		0.02		

**Table 2:** Frequency of electrocardiographic changes in patients with primary hyperthyroidism by gender

Gender	Sinus tachycardia		Atrial fibrillation		Total
	Yes	No	Yes	No	(n=103)
Male	14 (73.7%)	5 (26.3%)	0 (0%)	19 (100%)	19 (18.44%)
Female	48 (57.1%)	36 (42.9%)	12 (14.3%)	72 (85.7%)	84 (81.56%)
X^2^	1.76		3.07		
**p-value**	0.18		0.08		

## Discussion

It is well known that one of the main complications of thyrotoxicosis is heart disease, including heart rhythm abnormalities. Thyroid hormones are known to affect the cardiovascular system both directly and indirectly [9] and results in increased cardiac contractility, enhanced cardiac output and reduced systemic vascular resistance. Incidence of thyrotoxic heart disease in sub-Saharan Africa ranged from 6.2-8% [10-12].

Incidence of cardiac disease in thyrotoxicosis is 27% with male to female ratio of 1:5 [5], frequency of hypertension 53%, arrhythmia 25% and heart failure 42% [6]. Angina has been reported in 20% of the patients with thyrotoxicosis [4]. Several studies have reported that prolong QT interval is associated with increased risk of ventricular dysrrhythmia and sudden death [4]. Heart is one of the main target organs of thyroid hormone and its morphology and function are investigated by means of standard 12-leads ECG [7]. In this study only two ECG changes were recorded sinus tachycardia and atrial fibrillation.

Though cardiac complications of thyrotoxicosis had been reported to be more common in the elderly [13] but Nigerians had reported contrary to this observation [10-12]. The mean age of occurrence of cardiac complications of thyrotoxicosis as seen from this report is 40.8 years. The mean age of this group of people in this report though lower than that reported by Famuyiwa [10] is comparable to the more recent report by Danbauchi *et al.* [14]. In this report, cardiac complications of thyrotoxicosis were noted 30.09±5.57 years. Out of 103 cases, 19(18.45%) were male and 84(81.55%) were female. In Ogbera *et al.*, [5] study the Male: Female ratio of the subjects with thyrocardiac disease was 1:5.

Thyrotoxicosis is associated with arrhythmias; of which atrial fibrillation is the most commonly encountered arrhythmia [14]. The incidence of atrial fibrillation ranges from about 10-21% in patients with thyrotoxicosis [15], compared with 0.4% in the overall adult population [16, 17]. Though atrial fibrillation has been noted to occur more commonly in males with thyrotoxicosis [18, 19], this report shows a female preponderance which is in line with previous Nigerian reports [10, 20]. In this study frequency of electrocardiographic in term of sinus tachycardia was observed in 60.19% (62/103) patients and atrial fibrillation was 11.65 (12/103) cases.

Similar result was also observed in Rotman-pikieln [21]. In this study sinus tachycardia and atrial fibrillation occurred in 65.5 and 15.5% of the patients, more common in those with hyperthyroidism. Thyroid hormones affect cardiovascular system by increasing stroke volume and heart rate [22]. Excessive thyroid hormones are linked to many heart diseases such as angina, myocardial infarction, arrhythmia and sudden death [23]. The possible mechanisms of coronary occlusion with thyrotoxicosis include: significant underlying coronary atherosclerosis, direct damage to coronary artery and coronary embolization [24].

Several hypothesis have been proposed for the mechanism of thyroid hormone and coronary artery spasm. The basic idea is that a higher sensitive state of coronary artery to vasoconstrictive agents and a decreased level of vasodilator under thyrotoxic state [25]. In additional, coronary spasm produces a higher chance of atherosclerotic events owing to thrombus formation accelerating and fibrinolysis delaying. Thyrotoxicosis also leads to a hypermetabolic state and causes imbalance between blood supply and oxygen demand, resulting in cardiac symptoms [26].

There have been only a few cases reported for hyperthyroidism related coronary spasm. The diagnosis of hyperthyroidism may be overlooked or delayed in patients without typical symptoms. Lee *et al.* [27] reported a patient who complained of chest pain without specific symptoms of thyrotoxicosis. She was treated with emergent coronary artery bypass graft surgery due to ostial stenosis of coronary arteries on angiography. The patient had thyrotoxic storm postoperatively.

## Conclusion

In this study frequency of electrocardiographic in term of sinus tachycardia was high. This report has emphasized the importance of thyrotoxicosis as a cause of cardiac morbidity and mortality in patients with thyrotoxicosis hence cardiologist should lower down the threshold of diagnosing hyperthyroidism. These cardiac complications are readily reversible if there is timely optimal treatment offered.

### What is known about this topic

- ECG changes in hyperthyroidism are already known;
- Consequences of cardiac problems secondary to hyperthyroidism.

### What this study adds

- This is the first study done in our country about the ECG trends in patient suffering from hyperthyroidism;
- Cardiologist should lower there threshold of diagnosing hyperthyroidism in patient with such ECG changes.

## Competing interests

All authors declare no competing interests.

## References

[1] Lin JW, Lian WC, Lin Tk (2010). Coronary vasospasm associated with hyperthyroidism. Acta cardiol Sin.

[2] Zhang Y, Post WS, Cheng A, Blasco-Colmenares E, Tomaselli GF, Guallar E (2013). Thyroid hormones and electrocardiographic parameters: finding from the third national health and nutrirtion examination survey. PloS One.

[3] Kuang XH, Zhang SY (2011). Hyperthyroidism-associated coronary spasm: a case of non-ST segment elevation myocardial infarction with thyrotxicosis. J of geriatric cardiol.

[4] Filipescu D, Calugareanu A, Luchian M, Maricina I, Ghenu O, Marin S, Moldovan H (2009). Fatal myocardial infarction secondary to thyrotoxicosis: case report. Acta Endocrinol.

[5] Ogbera AO, Fasanmade O, Isiba A (2007). The scope of cardiac complications of thyrotoxicosis in lagos, Nigeria. Pak J Med Sci.

[6] Pikielny PR, Borodin O, Zissin R, Abramof RN, Levey Y (2008). Newly diagnosed thyrotoxicosis in hospitalized patients: clinical characteristics. QJ Med.

[7] Biondi B, Palmieri EA, Fazio S, Cosco C, Nocera M, Sacca L, Filetti S (2000). Endogenous subclinical hyperthyroidism affects quality of life and cardiac morphology and function in young and middle-aged patients. J Clin Endocrinol Metab.

[8] Iskandar SB, Jordan RM, Peiris AN (2007). Electrocardiographic abnormalities and endocrine disease-part a: hyperthyroidism. Tenn Med.

[9] Common K, Milburn KS, Cawood TS, Crozier I (2012). Coronary artery spasm due to thyrotoxicosis. NZ med J.

[10] Czarkowski M, Hilgertner L, Powalowski T, Radomski D, Mikulska M (2005). Is the resistance of large conduit arteries also decreased in thyrotoxic patients with Grave's disease?. Thyroid.

[11] Famuyiwa OO (1987). Cardiac disease in Nigerians with thyrotoxicosis. Tropical Cardiology.

[12] Olurin EO, Itayemi SO, Oluwasanmi Jo, Ajayi OO (1973). The pattern of thyroid gland diseases in Ibadan, Nigeria. Nigerian Med J.

[13] Nkoua JL, Mbam B, Bandoho-Mambo A, Aba G, Bouramoue CH Thyrotoxic heart disease; incidence, causes and clinical characteristics. A review of 20 cases. La Sante Tropicale sur Internet. Medicine d'Afrique Noire.

[14] Klein I, Ojamaa K (2001). Thyroid hormone and the cardiovascular system. New Eng J Med.

[15] Malvinder SP (2005). Thyrotoxic atrial fibrillation. Med Gen Med.

[16] Yuen RWH, Gutteridge DH, Thompson PL, Robinson JS (1979). Embolism in thyrotoxic atrial fibrillation. Med J Austr.

[17] Ostrander LD, Brandt RL, Kjelsberg MO, Epstein FH (1965). Electrocardiographic findings among the adult population of a total natural community, Tecumseh, Michigan. Circulation.

[18] Freedberg AS, Papp JG, Vaughan WEM (1970). The effect altered thyroid state on atrial intracellular potentials. J Physiol (Lond).

[19] Shimizu T, Koide S, Yoshimura Noh K, Sugino K, Ito H, Nakazawa H (2002). Hyperthyroidism and the Management of Atrial Fibrillation. Thyroid.

[20] Sandler G, Wilson GM (1959). The nature and prognosis of heart disease in thyrotoxicosis. QJ Med.

[21] Kolawole BA, Balogun MO (2001). Thyrotoxicosis and the heart: a review of the literature. Nig J Med.

[22] Klein I, Ojamaa K (2001). Thyroid hormone and the cardiovascular system. N Eng J Med.

[23] Lin TH, Su HM, voon WC (2005). Unstable angina with normal coronary angiography in hyperthyroidism: a case report. Kaohsiung J Med Sci.

[24] Resnekov L, Falicov RE (1977). Thyrotoxicosis and lactate-producing angina pectoris with normal coronary arteries. Br Heart J.

[25] Jung CH, Rhee EJ, Shin HS (2008). Higher serum free thyroxine levels are associated with coronary artery disease. Endocrine J.

[26] Yun KH, Jeong MH, Oh SK (2007). Relationship of thyroid stimulating hormone with coronary atherosclerosis in angina patients. Int J Cardiol.

[27] Lee SM, Jung TS, Hahm JR (2007). Thyrotoxicosis with coronary spasm that required coronary artery bypass surgery. Internal Med.

